# Two-Way Reversible Shape Memory Behavior of Chitosan/Glycerol Film Triggered by Water

**DOI:** 10.3390/polym15102380

**Published:** 2023-05-19

**Authors:** Shuozi Li, Hu Lyu, Yujia Wang, Xianzhi Kong, Xiangxian Wu, Lina Zhang, Xiaojuan Guo, Dawei Zhang

**Affiliations:** 1 Engineering Research Center of Advanced Wooden Materials, Ministry of Education, Northeast Forestry University, Harbin 150040, China; 15387045795@163.com (S.L.); wangyujia990508@163.com (Y.W.); w18773337677@163.com (X.W.); 13853065099@163.com (L.Z.); gxj15776632498@163.com (X.G.); 2 Institute of Petrochemistry, Heilongjiang Academy of Sciences, Harbin 150036, China; 15546002614@163.com

**Keywords:** reversible shape memory, chitosan, composite film, hydrogen bonds

## Abstract

Reversible shape memory polymers (SRMPs) have been identified as having great potential for biomedical applications due to their ability to switch between different shapes responding to stimuli. In this paper, a chitosan/glycerol (CS/GL) film with a reversible shape memory behavior was prepared, and the reversible shape memory effect (SME) and its mechanism were systematically investigated. The film with 40% glycerin/chitosan mass ratio demonstrated the best performance, with 95.7% shape recovery ratio to temporary shape one and 89.4% shape recovery ratio to temporary shape two. Moreover, it shows the capability to undergo four consecutive shape memory cycles. In addition, a new curvature measurement method was used to accurately calculate the shape recovery ratio. The suction and discharge of free water change the binding form of the hydrogen bonds inside the material, which makes a great reversible shape memory impact on the composite film. The incorporation of glycerol can enhance the precision and repeatability of the reversible shape memory effect and shortens the time used during this process. This paper gives a hypothetical premise to the preparation of two-way reversible shape memory polymers.

## 1. Introduction

Shape memory polymers, which have been widely used as a kind of smart material, can recover from the presupposed temporary shape to the permanent shape upon external stimulus [1,2,3,4]. However, the unidirectional nature of one-way shape memory deformation had restricted the application of materials in various fields. Derived from it, the researchers’ interest has gradually focused on the new reversible shape memory polymers (RSMPs), which could achieve the shape interconversion between two shapes while receipting the external stimuli [5,6,7,8].

Currently, the thermo-responsive bidirectional RSMPs have occupied the research’s highlights [9,10,11,12,13]. For semi-crystalline polymers, the directional crystallization of anisotropic polymer networks would be responsible for the thermal-triggered two-way SME [14,15,16]. Due to the relevance between the thermal effect and mechanical behaviors of semi-crystalline SMPs, the effect of thermal stimuli on the SMPs within a certain temperature range could be anticipated and controlled.

Solution actuation [17,18,19,20,21,22], as a triggering pattern through changing the polymer structure since Huang [23], develops relatively slower because the effect of the solution on the RSMPs is all-directional and complex. The solvent-driven shape memory effect can be classified into two modes: water-driven and organic solvent-driven [24,25,26]. Various solvents can trigger different shape recovery responses, similar to the temperature memory effect, and the use of diverse solvents can enable multi-staged and multiple shape memory recovery mechanisms [27,28]. Therefore, how to improve the recovery speed, accuracy and stability of RSME of polymers, especially of natural polymer materials upon the solution, has become an investigated hotspot. The SME of shape-memory materials is always associated with their crystallization properties [29]. The entry of solution molecules swells the molecular chains in amorphous regions, and the movement of the segments eventually leads to macroscopic deformation, which can be achieved through the disruption and reforming of intermolecular hydrogen bonds [30,31].

Chitosan (CS), as the only biodegradable cationic polysaccharide originated from the partial deacetylation of chitin from the shells of crustaceans, has attracted great research interest due to its wide availability, biocompatibility and biodegradability [32,33,34,35,36]. The use of chitosan-based biomaterials is integral in skin tissue engineering primarily [37]. Its antimicrobial and hemostatic properties allow chitosan to promote wound healing and prevent infections [38,39]. The hydrogen bonds formed between the chain segments benefit from the amino and hydroxyl groups; the protonation of amino groups allows electrostatic interactions, which could be conducive to the film formation in an acidic medium [40,41]. 

In this paper, chitosan/glycerol (CS/GL) films were prepared, and a novel curvature method was employed to characterize the shape programming rate and recovery rate of RSMPs. Glycerol (GL) is dispersed within chitosan molecules primarily through hydrogen bonding and the van der Waals force. This dispersion enhances the structure of the chitosan network, allowing for a more stable CS/GL complex in solution. Compared to polymers, such as polyvinyl alcohol [42], glycerol’s smaller molecular size results in improved solubility and biocompatibility. The RSME was analyzed by various characterization techniques including dynamic mechanical analysis (DMA), thermogravimetric analysis (TGA), Fourier transform infrared (FTIR), differential scanning calorimetry (DSC) and X-ray diffraction (XRD). Through the implementation of a water–air bidirectional triggering mode, we demonstrated the ability of RSMPs to exhibit completely reversible shape memory behavior under conditions of water absorption and loss, with increased glycerol content resulting in a greater shape recovery rate and a shorter response time of RSMPs. Through our investigation, it was found that the stronger the mobility of chitosan chains, the stronger the RSME. The binding mode of water molecules was also closely related to different stages of RSME. Furthermore, we emphasized the key roles of intermolecular hydrogen bonding in shaping the mechanical properties of RSME.

## 2. Materials and Methods

### 2.1. Materials

CS (degree of deacetylation 90.3%, viscosity 100 mPa·s) was purchased from Zhejiang Golden Shell Pharmaceutical Co. (Zhejiang, China). GL (purity 99%) was commercially available from Tianjin Hengxing Chemical Reagent Co. (Tianjin, China). Acetic acid (purity 99.5%) and NaOH (purity 99.0%) were both obtained from Tianjin Kermel Chemical Reagent Co. (Tianjin, China). Distilled water was bought from Harbin Wenjing (Harbin, China) distilled water factory. Ethanol (purity 99.7%) was obtained from Tianjin Zhiyuan Chemical Reagent Co. (Tianjin, China). Unless otherwise stated, all the materials were of analytical grade and all the materials were used without further treatment.

### 2.2. Preparation of CS/GL Film

**Figure 1:**
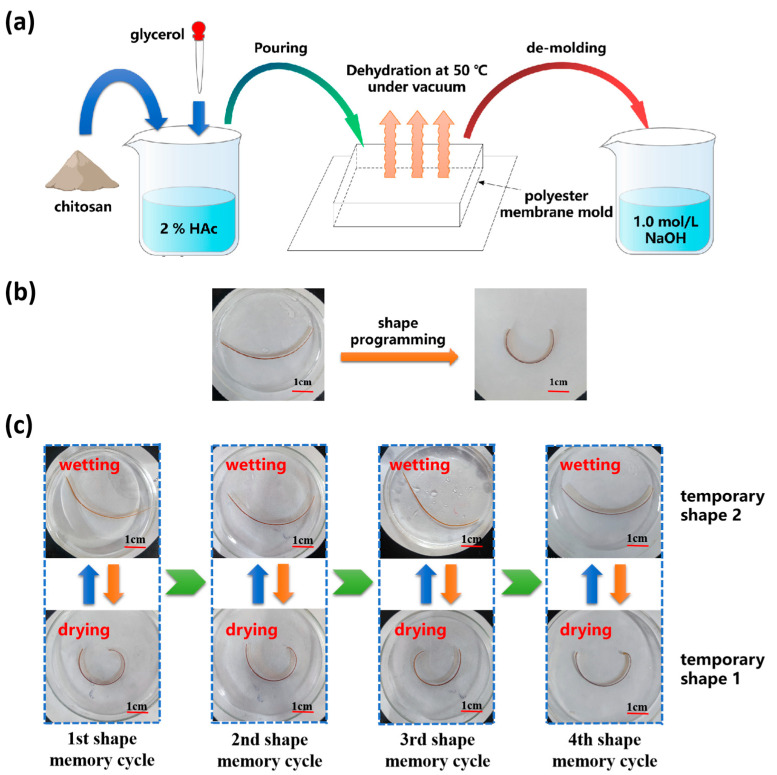
Preparation method of CS/GL films with RSME and the shape memory process of RSMPs. (**a**) Preparation method of CS/GL films; (**b**) thermal programming of RSMPs (under 40% GL mass ratio) to obtain a curve shape (temporary shape 1); and (**c**) RSME between temporary shape 1 shape and temporary shape 2 can be repeated for many times (RSMPs under 40% GL mass ratio).

Figure 1a. shows the preparation method of CS/GL films. The powdered CS was dissolved in 2% weight percent acetic acid and stirred evenly to obtain 3% wt CS solution. Glycerol was added to the CS solution and stirred for 1 h to obtain the 10%, 20%, 30%, and 40% GL/CS (mass ratio) solutions. The resulting solutions were poured into a homemade film-shaping mold and allowed to dry at 50 °C and −0.09 MPa for 72 h to fabricate uniform CS/GL films.

### 2.3. Shape Memory Test

To prepare RSMPs for testing, rectangular samples with a width of 3 mm and a length of 50 mm were cut from the prepared films. These samples were immersed in a 1.0 mol/L NaOH solution for 30 s and then washed with distilled water. The prepared samples were thermally programmed on the inner surface of the mold to obtain its temporary shape 1 in an oven at 80 °C for 1 h, as shown in Figure 1b.

**Figure 2:**
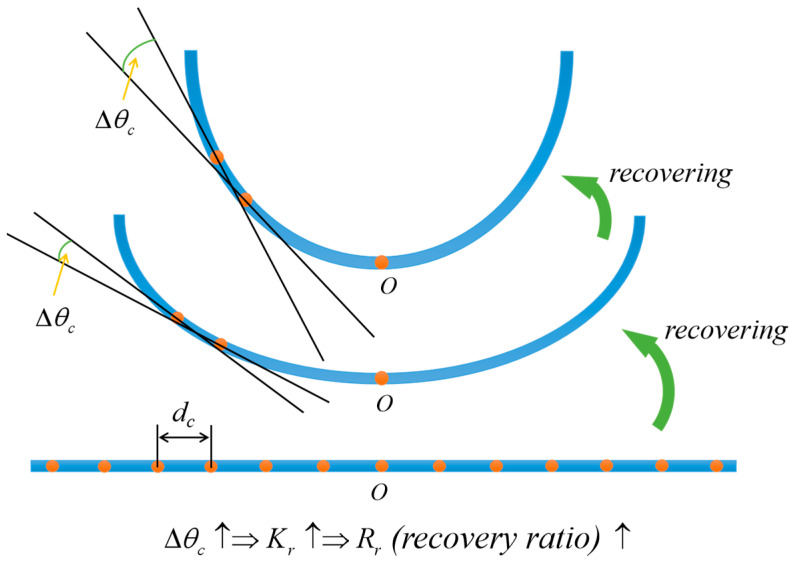
The recovery ratio measured by curvature was used to characterize differences in the degree of deformation across different shape memory processes. Curvature-determining method used for testing RSME.

A curvature determination method was utilized to characterize and evaluate the RSME in the process of shape programming and shape recovery, as depicted in Figure 2. The shape programming ratio ($R_{p}$) was employed to evaluate the degree of thermal programming of the material, which was determined by dividing $\bar{K_{p}}$ by $\bar{K_{p0}}$:

(1) $$R_{p}=\frac{\bar{K_{p}}}{\bar{K_{p0}}}\times 100\%$$

where $\bar{K_{p}}$ represents the mean curvature of the sample after thermal molding, while $\bar{K_{p0}}$ denotes the inner surface curvature of the mold used for fixing the sample.

After thermal shape programming, the sample was cooled adequately at room temperature and placed in water to elicit a response. After a certain period, the sample obtained temporary shape 2 (flattened shape). The shape recovery ratio of temporary shape 2 ($R_{r2}$) was determined by $\bar{K_{r0}}$ and $\bar{K_{r2}}$:

(2) $$R_{r2}=(1-\frac{\left|\bar{K_{r2}}-\bar{K_{r0}}\right|}{\bar{K_{r0}}})\times 100\%$$

where $\bar{K_{r0}}$ represents the mean curvature of the sample during the first extension in water and $\bar{K_{r2}}$ represents the average curvature when temporary shape 2 was obtained again after undergoing the first shape memory cycle.

The sample with temporary shape 2 was removed from the water environment and exposed to air. After the sample underwent a dehydration process in the air (the abbreviation “in air” is used later in the text), it regained its temporary shape 1, determined during thermal programming. During this process, the rate of water loss depends to some extent on the temperature and air humidity. In this study, the samples were exposed to the air for dehydration in a closed laboratory with a temperature of 25 °C and a relative humidity of 41%. At this point, the shape recovery ratio $R_{r1}$ was determined by dividing $\bar{K_{r1}}$ by $\bar{K_{p}}$:

(3) $$R_{r1}=\frac{\bar{K_{r1}}}{\bar{K_{p}}}\times 100\%$$

where $\bar{K_{r1}}$ denotes the mean curvature of the sample after shape recovery to temporary shape 1.

During an entire cycle of shape memory transformation, the deformation state of the RSMP was recorded using a camera every 3 s. One shape recovery cycle is defined as follows: the temporary shape 1 transforms into the temporary shape 2 in response to water stimulation, and then the temporary shape 2 transforms back to the temporary shape 1 after dehydration in the air. The material’s training through thermal programming is independent of the shape recovery cycle. In this paper, the shape recovery time is defined as the time required for the material to undergo directional shape memory deformation and deform to another stable shape after being stimulated by the external environment from a stable shape. The judgment method for stable shape is: if the deviation between the shape recovery ratios measured within 60 s after this state is less than 0.2%, the material in this state is determined to have a stable shape.

Methods to calculate the mean curvature of RSMPs are shown below. Set a positive integer n greater than 3; the RSMPs sample was divided into (*n* + 1) segments with equal lengths by n points. The distance between adjacent points was denoted as *d* and the angles between tangents ($\Delta \theta_{c}$, *c* = 1, 2, 3 … *n*) were calculated. The mean curvature was defined as follows:

(4) $$\bar{K}=\frac{1}{n}\sum_{c=1}^{n}\frac{\Delta \theta_{c}}{d}\;(c=1,\;2,\;3\;\ldots \;n)\;$$

It should be noted that the larger the value of *n*, the more significant the measured mean curvature is. In this paper, the value of n was uniformly set to 9.

### 2.4. Characterization and Measurements

Free water content and thermal stability of samples were performed on a thermal analyzer (TG 209 F3, Netzsch) under an N_2_ flow at a heating rate of 10 °C/min. Dynamic mechanical behaviors of samples were carried out using a DMA-242 E at a heating rate of 5 °C/min with 10 μm maximum tensile strain from −30 °C to 100 °C. Fourier transform infrared (FTIR) spectra (TENSORIIBruker, Ettlingen, Germany) were selected to characterize the chemical structure of RSMPs and changes in hydrogen bonding interactions at different shape memory stages, covering the range of 4000–400 cm^−1^ with a resolution of 4 cm^−1^. The crystallinity of RSMPs at different stages of RSME was investigated by X-ray diffraction (XRD) analysis performed with a Rigaku D/max 2200 VPC (Tokyo, Japan). XRD patterns were examined within the diffraction angle from 5° to 65° with a scanning speed of 5°/min. Differential scanning calorimetry (DSC) analysis was conducted on a Netzsch 204 DSC instrument under an N_2_ flow. The samples were heated up from 30 °C to 200 °C with the warming rate 5 °C/min throughout the whole process.

## 3. Results and Discussion

### 3.1. Two-Way Reversible Water-Triggered Shape Memory Behaviors

**Figure 3:**
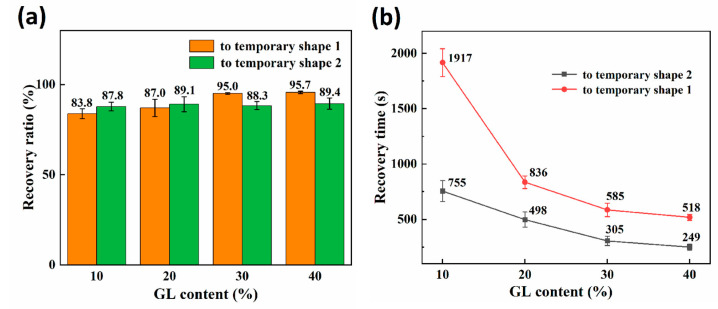
Characterization of the two shape recovery processes in a shape memory cycle. (**a**) The recovery ratio of the reversible shape memory polymers (RSMPs) under various GL ratios; (**b**) the recovery time of RSMPs under various GL ratios.

As illustrated in Figure 3a, the shape memory effect of RSMPs with 10% and 20% GL ratio was not entirely satisfactory, evidenced by $R_{r1}$ values of only 83.8% and 87%, respectively. However, when increasing the GL ratio to 30% and 40%, the $R_{r1}$ value could reach 95.0% and 95.7%, indicating that the materials have excellent RSME at the macroscopic level. In contrast, there was no significant difference in the $R_{r2}$ value of RSMPs undergoing a transformation from temporary shape one to temporary shape two for various GL concentrations. Remarkably, as shown in Table 1, irrespective of varying glycerol content, the $R_{p}$ value of the materials exceeded 98%, cementing CS’s position as an exceptional matrix material for shape memory materials. As shown in Figure 3b, the shape recovery time of RSMPs during both stages exhibited an increasing reduction with a greater GL ratio, thereby affirming an escalated responsiveness to environmental stimuli. It is noteworthy that the transition time of RSMPs to temporary shape one was longer than that to temporary shape two, which mainly depends on the rate of water absorption and desorption.

The CS/GL composite film exhibits remarkable reversibility in the process of bidirectional shape memory, which is manifested by consistent recovery time and consistent recovery ratio across multiple shape memory cycles (Figure 1c). Meanwhile, this reversible bidirectional shape memory process can be forcibly interrupted at any stage and redriven in both directions (to temporary shape one or temporary shape two). The thermally programmed shape (temporary shape one) of the composite film was obtained during water loss in air, regardless of whether the material was fully soaked in water during the previous process. Similarly, regardless of whether the composite film was completely dehydrated in air, it eventually recovered to temporary shape two in the aqueous solution. Mechanistically, the polymer deformation process could be attributed to a relaxation behavior [43,44], which was always synchronized with real-time changes in the polymer network for RSME. The high degree of reversibility of RSME was largely attributed to the reversibility of the structural changes in polymer networks.

### 3.2. Thermogravimetric (TG) Analysis

**Figure 4:**
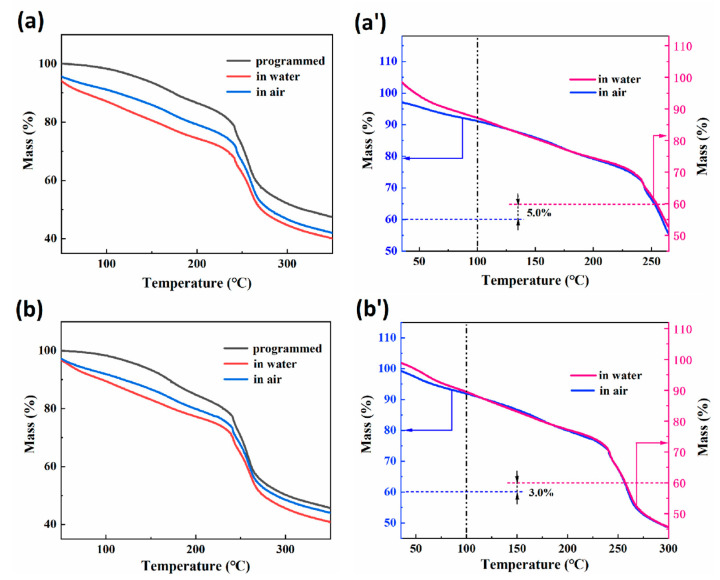
Results of TG analysis after shape programming and in the first shape memory cycle. (**a**) TG curves of 30-RSMPs; (**a′**) parallel determination of the two curves from (**a**); (**b**) TG curves of 40-RSMPs; (**b′**) parallel determination of the two curves from (**b**).

Thermogravimetric (TG) curves of RSMPs with a 30% GL ratio (named 30-RSMPs) at different stages of RSME (after shape programming and in the first shape memory cycle) are shown in Figure 4a. Figure 4a′ represents two curves from Figure 4a (in water and in air), with the TG curve in air having the intercept corresponding to 5% of water content subtracted. Figure 4b displays the TG curves of samples with a 40% GL ratio (named 40-RSMPs) at different RSME stages; Figure 4b′ is the curve obtained by adjusting the intercept of 3% water content between the two curves (in water and in air) shown in Figure 4b, respectively. It can be observed that the curves in Figure 4 follow the typical pattern of thermal degradation of natural polymers [45,46]. Most of the free water would be removed in the heating process (<100 °C), while the dehydration of combined water was a gradual process during the heating process. When the temperature reached 250 °C, the curve dropped sharply, indicating the significant thermal degradation of RSMPs. Comparing Figure 4a′,b′, it could be observed that after compensating for the difference in water content, the TG curves of the water-absorbed sample and the dehydrated sample (>100 °C) are almost identical, indicating that the water triggering the RSME of the composite films exists in the form of free water. Moreover, the difference in water content has no influence on the internal structure of the material. The change in crystallinity was usually accompanied by the change of thermal stability for natural polymer materials [47,48], and the parallelism of TG curves between two shapes indicated that the entry and exit of water did not affect the crystallization region of CS. It is evident that the water molecules lost by RSMPs between temporary shape one and temporary shape two did not bind with chitosan, but rather formed hydrogen-bonded chains to occupy the gaps in the chitosan chain segments, which mainly consisted of free water. In contrast, the TG curve of the thermally-programmed material did not exhibit such “parallelism” with the temporary shape one and temporary shape two curves. Therefore, it can be confirmed that the water molecules absorbed by the material during the transition from thermal programming to the shape memory period were linked with chitosan through hydrogen bonding, which is referred to as bound water.

### 3.3. Differential Scanning Calorimetry (DSC) Analysis

**Figure 5:**
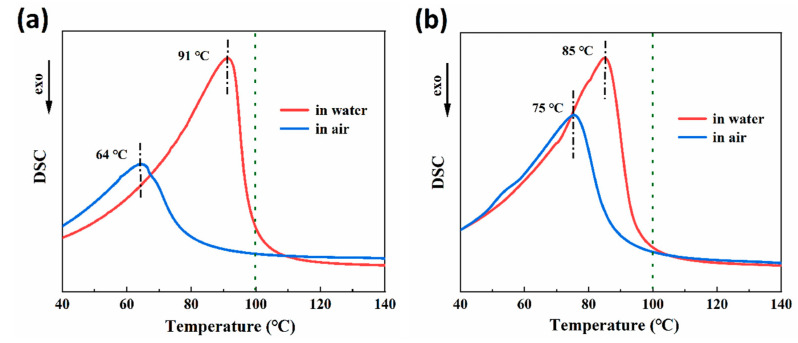
Differential scanning calorimetry (DSC) analysis in the first shape memory cycle. (**a**) DSC curves of 30-RSMPs in the first shape memory cycle; (**b**) DSC curves of 40-RSMPs in the first shape memory cycle.

Figure 5a,b display the results of DSC tests conducted on 30-RSMPs and 40-RSMPs during the first shape memory cycle. The 30-RSMPs DSC curve exhibits a significant endothermic peak at 91 °C (in water) and 64 °C (in air), while the 40-RSMPs DSC curve exhibits similar peaks at 85 °C (in water) and 75 °C (in air). The peak difference for 40-RSMPs is only 10 °C, which is significantly less than 30-RSMPs’ 27 °C, thus confirming that the recovery time (to temporary shape one and to temporary shape two) for 40-RSMPs is faster than for 30-RSMPs. The shape recovery speed is regulated by the glycerol content in the film. With an increase in the glycerol content, the water absorption and water loss is fast, thus recovery speed of the film is increased. The absorption or loss water was in the form of free water; the results observed were consistent with the findings from the thermogravimetric (TG) analysis.

### 3.4. Dynamic Mechanical Behaviors of CS/GL Composite Films

**Figure 6:**
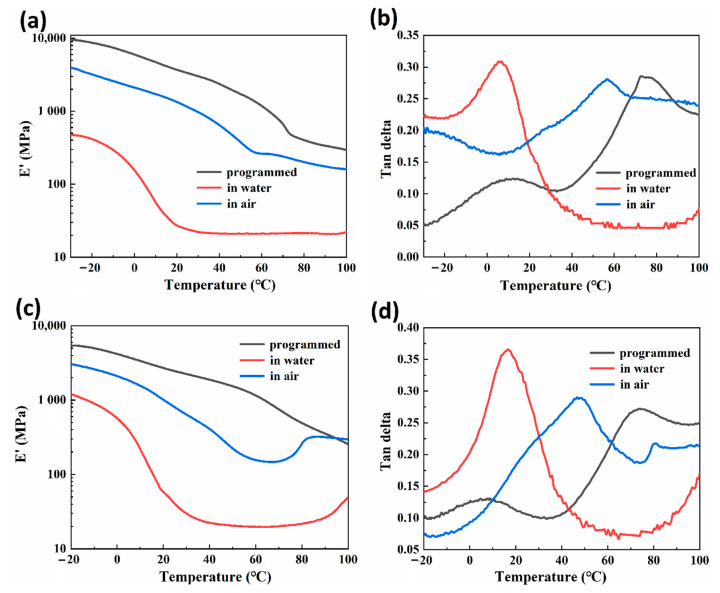
Dynamic mechanical analysis after shape programming and in the first shape memory cycle. (**a**) Storage modulus curves of 30-RSMPs; (**b**) tanδ curves of 30-RSMPs; (**c**) storage modulus curves of 40-RSMPs; and (**d**) tanδ curves of 40-RSMPs.

Figure 6a,b show the results of the DMA (dynamic mechanical analysis) test of 30-RSMPs at different stages of RSME (after shape programming and in the first shape memory cycle), while Figure 6c,d shows the results of 40-RSMPs at different stages of RSME. In Figure 6a,c, the storage moduli gradually decreased with the increase in temperature. Compared with these two groups, the storage modulus was negatively correlated with water content, which proved that the water triggering of RSMP could be interpreted as a process of reducing storage moduli. As shown in Figure 5b, the storage moduli of CS networks exhibit a sharp decrease at temperatures corresponding to this transition caused by water molecule diffusion. In an environment with air exposure, water molecules gradually diffuse from the material into the surrounding air, causing a reduction in the available space for polymer chain mobility. This results in increased entanglements between the polymer chains, which in turn leads to a corresponding increase in the strength of the samples. Accordingly, the secondary transition temperature of RSMPs after programming, immersing in water, and exposing to air were T_p_ = 76.0 °C, T_w_ = 6.1 °C and T_a_ = 56.7 °C [49,50], as well as the transition temperature in Figure 5d being T_p_ = 74.2 °C, T_w_ = 16.9 °C and T_a_ = 47.3 °C, respectively. The secondary transition temperature decreased when the RSMP was immersed in water while increased in air condition. This suggests that the triggering effect of water molecules on shape memory behaviors occurs within the amorphous region of CS, which is likely due to the presence of reversible hydrogen bonds [51,52,53]. When water infiltrates the CS molecules, the hydrogen bonds between the CS chains are disrupted, causing the chain segments to become mobile and resulting in an earlier appearance of the tanδ peak. After water actuation, the tanδ peak of 40-RSMPs was higher than that of 30-RSMPs. In addition, the tanδ peak of both RSMPs with 30% and 40% GL ratio increased after the water immersion. The tanδ peak in 30-RSMPs increased from 0.285 to 0.307 and from 0.272 to 0.364 in 40-RSMPs. Under the premise that 40-RSMPs had a shorter water absorbing time, the swelling effect of water molecules is more effective on the 40-RSMPs, thus significantly increasing the tanδ peak.

### 3.5. Fourier Transform Infrared (FTIR) Spectra

**Figure 7:**
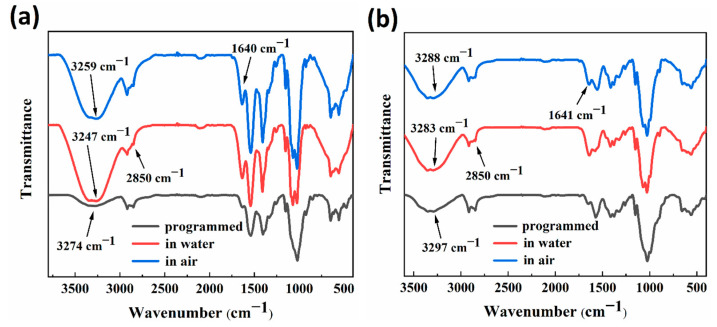
FTIR test spectra after shape programming and in the first shape memory cycle. (**a**) FTIR spectra of 30% GL ratio RSMPs; (**b**) FTIR spectra of 40% GL ratio RSMPs.

Figure 7a,b showed the FTIR test spectra of RSMPs at different stages of RSME (after shape programming and in the first shape memory cycle) with 30% and 40% GL ratio. According to the curve analysis in the two plots, for the programmed RSMPs, the two absorption peaks at 3274 cm^−1^ and 3297 cm^−1^ were assigned to the overlapping results of O−H and N−H stretching vibrations. In response to water stimulation, the overlapping peak moved in low wavenumbers to 3247 cm^−1^ and 3283 cm^−1^, respectively. Moreover, when the SMP responded in the air environment, the overlapping peaks moved again to the high wavenumber of 3259 cm^−1^ and 3288 cm^−1^ but were still lower than the programmed wavenumber. This was strong evidence for the formation of hydrogen bond-based supramolularly reversible cross linking inside RSMP networks. The absorption peak at 2850 cm^−1^ was assigned to the C−H of methylene at the carbon atom at position 6 (C6) and its intensity decreased delicately with the formation of hydrogen bonds between water and hydroxyl groups at C6. The elongation vibration peak of C=O corresponded to the RSMPs of 1640 cm^−1^ and 1641 cm^−1^ in two plots, with a significantly enhanced peak strength after increasing the water content, which was also associated with the base energy to form reversible hydrogen bonds with water molecules. Therefore, the process of hydrogen bond re-establishment and the swelling effects of solution molecules could be important sources of reproducibility and reversibility of RSME.

### 3.6. X-ray Diffraction (XRD) Analysis

**Figure 8:**
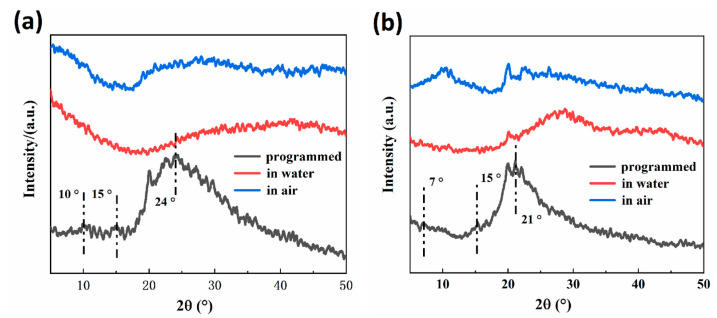
XRD spectra of RSMPs after shape programming and in the first shape memory cycle. (**a**) XRD spectra of 30-RSMPs at different stages of RSME; (**b**) XRD spectra of 40-RSMPs at different stages of RSME.

The crystalline properties (after shape programming and in the first shape memory cycle) at different stages of RSME of 30-RSMPs and 40-RSMPs were investigated through XRD analysis. As shown in Figure 8a, diffraction peaks were observed at around 2θ = 10°, 2θ = 15° and 2θ = 24°, while at around 2θ = 7°, 2θ = 15° and 2θ = 21° in Figure 8b, respectively, indicating the semi-crystalline nature of the CS. 

The crystallinity of the 30-RSMPs and 40-RSMPs was found to be 39.1% and 35.7% (shown in Table 2), respectively, which represents the highest achievable degree of crystallinity for RSMPs without free water. However, as the material absorbs water, the intensity of these diffraction peaks significantly reduces, resulting in a smoother diffraction pattern and a broader XRD spectrum presenting a smooth, bun-like peak. Therefore, determining the crystallinity of 30-RSMPs and 40-RSMPs at this stage is no longer meaningful. Upon exposure to air, the diffraction peaks gradually became narrower and more pronounced, and the crystallinity of RSMPs increased to some extent. These findings suggest that water content is an important factor that affects the crystalline properties of the RSMPs. The entry of water molecules disrupts the hydrogen bonding interactions between CS chains, promoting the migration of CS chain segments. As water molecules penetrate the gaps between chitosan chains, hydrogen bonds are formed between water molecules and CS chains, opening the pores in the gaps between chitosan chains, effectively enhancing the flexibility of polymer chains and reducing the crystallinity of RSMPs. It is noteworthy that the crystallinity of 30-RSMPs was consistently higher than that of 40-RSMPs, indicating the significant plasticizing effect of GL on CS. Increased GL content decreased the crystallinity of CS, promoting the motion of polymer chain segments, which is consistent with the experimental results obtained earlier.

**Figure 9:**
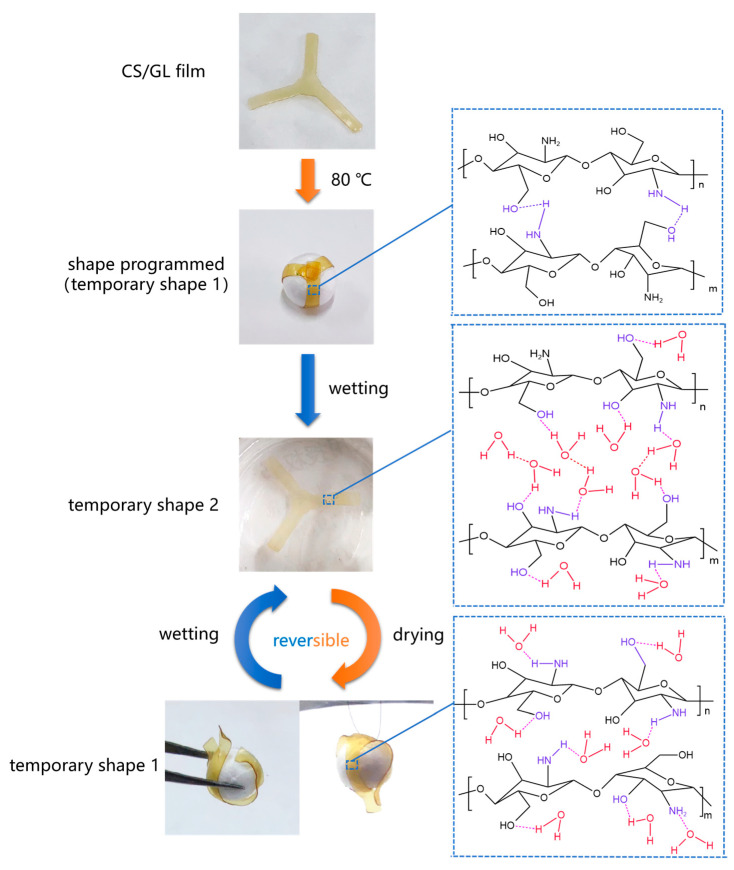
Schematic illustration of the reversible shape memory behavior of CS/GL films under 40% GL ratio triggered by water.

A schematic model is proposed through the above discussion to better understand the water triggered reversible shape memory mechanism of CS/GL films, as shown in Figure 9. RSMPs of a specific shape were thermally programmed by bending loading to obtain and store stress and fix it into a temporary shape one. At this point, chitosan segments are connected by hydrogen bonds and contain a small amount of bound water. When RSMPs are stimulated by water, they obtain temporary shape two and chitosan molecules absorb a large amount of water. The water disrupts the hydrogen bonds between chitosan–chitosan and forms new hydrogen bonds with the amino and hydroxyl groups of chitosan, which becomes bound water. At the same time, the water molecules form a hydrogen bond chain, swelling the chitosan matrix. This water exists in the form of free water. Afterward, if the RSMPs are placed in an air environment, the water will naturally diffuse and evaporate, and the RSMPs will regain their temporary shape one. Free water, which is swelling between chitosan segments, is evaporated and removed, and the stress stored between segments is released, causing them to arrange towards temporary shape one. After a shape memory cycle, the RSMPs can be dissolved in water and exposed to air to enter another shape memory cycle. During a shape memory cycle, temporary shape one can be further dehydrated to lose all free water and some bound water to obtain sufficient mechanical strength (such as grabbing and hanging a ball in Figure 9), but it needs to reabsorb enough bound water to re-enter the shape memory cycle.

## 4. Conclusions

This paper provides a comprehensive understanding of the reversible shape memory behavior of chitosan/glycerol (CS/GL) films triggered by water. The systematic investigation of the binding mode and mechanism between chitosan and water molecules in different shape memory stages sheds light on the intricate interplay between water and hydrogen bonding in the film. Specifically, when CS/GL films are thermally programmed at a specific shape and store stress, their CS segments are connected by hydrogen bonds and contain a small amount of bound water, leading to temporary shape one. Upon contact with water, the CS molecules absorb a large amount of water, disrupting the CS–CS hydrogen bonds and forming new CS–water hydrogen bonds with amino and hydroxyl groups. Furthermore, the free water molecules form a hydrogen bond chain, swelling the CS matrix. When the RSMPs are placed back in the air environment, the water naturally diffuses and evaporates, causing them to revert to temporary shape one, due to the release of the stress stored between the CS segments and further removal of free water. Moreover, the GL content was found to significantly impact the material’s shape memory performance, with higher concentrations improving the flexibility and response rate of the films. These findings not only contribute to our fundamental understanding of the shape memory behavior of CS/GL composite films but also hold promising implications for their potential application in various fields where reversible shape changes are desired.

## Figures and Tables

**Table 1:** Programming ratio of RSMPs under various GL ratios.

GL Content	Programming Ratio	Standard Deviation
10%	98.7%	0.21%
20%	98.8%	0.21%
30%	99.1%	0.25%
40%	98.9%	0.26%

**Table 2:** The crystallinity of RSMPs attached to the XRD spectra, “-” means the crystallinity that cannot be accurately calculated.

RSMPs	Crystallinity (Programmed)	Crystallinity (in Air)	Crystallinity (in Water)
30-RSMPs	39.1%	-	-
40-RSMPs	35.7%	-	-

## Data Availability

Not applicable.
